# Reliability of preoperative breast biopsies showing ductal carcinoma in situ and implications for non-operative treatment: a cohort study

**DOI:** 10.1007/s10549-019-05362-1

**Published:** 2019-08-06

**Authors:** Gurdeep S. Mannu, Emma J. Groen, Zhe Wang, Michael Schaapveld, Esther H. Lips, Monica Chung, Ires Joore, Flora E. van Leeuwen, Hendrik J. Teertstra, Gonneke A. O. Winter-Warnars, Sarah C. Darby, Jelle Wesseling

**Affiliations:** 1 Nuffield Department of Population Health, University of Oxford, Old Road Campus, Oxford, OX3 7LF UK; 2 Antoni van Leeuwenhoek – Netherlands Cancer Institute, Amsterdam, The Netherlands

**Keywords:** Ductal carcinoma in situ, DCIS, Breast cancer, Biopsy, Upgrading, Upstaging

## Abstract

**Purpose:**

The future of non-operative management of DCIS relies on distinguishing lesions requiring treatment from those needing only active surveillance. More accurate preoperative staging and grading of DCIS would be helpful. We identified determinants of upstaging preoperative breast biopsies showing ductal carcinoma in situ (DCIS) to invasive breast cancer (IBC), or of upgrading them to higher-grade DCIS, following examination of the surgically excised specimen.

**Methods:**

We studied all women with DCIS at preoperative biopsy in a large specialist cancer centre during 2000–2014. Information from clinical records, mammography, and pathology specimens from both preoperative biopsy and excised specimen were abstracted. Women suspected of having IBC during biopsy were excluded.

**Results:**

Among 606 preoperative biopsies showing DCIS, 15.0% (95% confidence interval 12.3–18.1) were upstaged to IBC and a further 14.6% (11.3–18.4) upgraded to higher-grade DCIS. The risk of upstaging increased with presence of a palpable lump (21.1% vs 13.0%, *p*_difference_ = 0.04), while the risk of upgrading increased with presence of necrosis on biopsy (33.0% vs 9.5%, *p*_difference_ < 0.001) and with use of 14G core-needle rather than 9G vacuum-assisted biopsy (22.8% vs 7.0%, *p*_difference_ < 0.001). Larger mammographic size increased the risk of both upgrading (*p*_heterogeneity_ = 0.01) and upstaging (*p*_heterogeneity_ = 0.004).

**Conclusions:**

The risk of upstaging of DCIS in preoperative biopsies is lower than previously estimated and justifies conducting randomized clinical trials testing the safety of active surveillance for lower grade DCIS. Selection of women with low grade DCIS for such trials, or for active surveillance, may be improved by consideration of the additional factors identified in this study.

**Electronic supplementary material:**

The online version of this article (10.1007/s10549-019-05362-1) contains supplementary material, which is available to authorized users.

## Background

The introduction of breast screening programmes has resulted in a dramatic increase in the annual incidence of ductal carcinoma in situ (DCIS), which currently accounts for approximately one fifth of screen-detected breast cancers in many countries worldwide [1–3]. Almost all women with DCIS receive either mastectomy or breast conserving surgery often with radiotherapy. Sometimes adjuvant hormonal treatment may also be prescribed. Since many DCIS lesions would never have become clinically significant in the absence of screening [4], this combination of therapies may constitute substantial overtreatment—especially since each treatment carries risks in addition to its intended benefits. Therefore, research in the field of DCIS management now focuses on finding ways to distinguish DCIS lesions requiring active treatment from those that can safely be left under surveillance and treated only if there is a change in morphology, grade, or invasive status [5, 6].

There is strong evidence that low grade DCIS, if it progresses, generally results in prognostically favourable, lower grade invasive breast cancer (IBC) [7]. Only the minority of screen-detected DCIS lesions will progress to IBC and the outcome for lower grade, early stage IBC is good. Therefore, randomized clinical trials are now underway to evaluate whether active surveillance of lower grade, screen-detected DCIS lesions is safe [8–10]. There are, however, concerns among healthcare providers and women with DCIS regarding participation in these trials. These concerns centre on three issues. First, a diagnosis of lower grade DCIS based on needle biopsy alone might underestimate the true extent of disease, as sometimes IBC or higher grade DCIS is found in the subsequent surgically resected specimen [11, 12]. Second, it is unclear whether inter-observer variation amongst pathologists in determining DCIS grade from preoperative biopsies may result in some women being considered inappropriately for non-operative management [13]. Third, it is unclear how successful the eligibility criteria used by the ongoing randomised trials of non-operative management in DCIS will be in selecting only women whose biopsies would not have been upstaged or upgraded if surgery had been performed [14]. These concerns are impacting participation in trials exploring the safety of active surveillance. They arise largely from earlier studies that reported the proportion of DCIS biopsies missing the presence of IBC as approximately one in four [15]. However, most of these studies did not exclude biopsies that were performed because IBC was already suspected.

Much of the existing literature is based on small, heterogeneous cohorts of women with DCIS diagnosed using small gauge biopsies and they often include women who presented symptomatically or with a mass-effect seen on mammography [15–17]. These factors limit generalizability to the population of women who may be considered for non-operative management today. Consequently, there is now a need for studies designed specifically to address concerns regarding the reliability of preoperative biopsy in DCIS when considering non-operative management. The present study evaluates the proportion of preoperative biopsies showing DCIS that were subsequently upstaged to IBC or upgraded to higher grade DCIS following pathological analysis of the surgically excised specimen, after exclusion of women already known to be at increased risk of invasive disease. It also identifies risk factors associated with upstaging or upgrading biopsy diagnoses of DCIS and it describes the extent to which interobserver variability affects preoperative DCIS grade ascertainment and the characteristics of the missed IBC component in upstaged biopsies. Finally, it evaluates the influence of the eligibility criteria of ongoing randomised trials on upgrade and upstage risks.

## Methods

### Material

Biopsies showing DCIS were selected through the Netherlands nationwide registry of histology and cytopathology records (PALGA) [18] and through the regional tumour registry at the Netherlands Cancer Institute—Antoni van Leeuwenhoek Hospital (NKI–AVL) (Supplemental Appendix 1). These preoperative biopsies were either performed at NKI–AVL or were taken at another hospital and sent for routine second opinion to NKI–AVL. NKI–AVL is a large specialist cancer centre that uses an integrated electronic patient record system to store clinical and radiological information. It also has a large pathology database containing information on the initial biopsy diagnosis of every woman referred to its breast cancer service, as well as the final pathological diagnosis of each woman with DCIS or IBC treated at NKI–AVL, thus allowing a retrospective cohort study to be conducted. The study was approved by the NKI–AVL ethical review board.

Biopsies were excluded if the initial biopsy did not show DCIS after reviewing the pathology reports, if lobular carcinoma in situ was reported, or when there was suspicion for, or evidence of, IBC. Biopsies were also excluded if either the initial biopsy or the final surgical specimen were not reviewed at NKI–AVL. When multiple biopsies were taken from the same lesion, only the biopsy with the highest DCIS grade was included. After these exclusions based on pathological factors, biopsies from women with clinically suspected (based on mammography and/or ultrasonography and/or clinical examination) or proven ipsilateral IBC elsewhere in the breast or in the lymph nodes, or with proven contralateral IBC for which neoadjuvant chemotherapy had been given, were excluded. Biopsies were also excluded if DCIS was discovered only after an MRI was performed or if only lymph node metastases were found and no breast invasion on final pathology.

### Data collection

Pathological information from the initial biopsy and from the surgically excised specimen was abstracted from the pathology records to identify women whose preoperative biopsy result was subsequently upstaged to IBC or upgraded to a higher grade of DCIS following surgery (Supplemental Table 1). Clinical and radiological information was abstracted from the electronic patient record system. To evaluate the tumour characteristics (size, grade and oestrogen receptor (ER) status) of the invasive component in upstaged cases, detailed review of the surgically excised specimens was undertaken.

#### Inter-observer analysis

Digital mammography was introduced at NKI–AVL in 2008. To examine the quality of the data extracted from the electronic records, all upstaged biopsies and a random sample of non-upstaged biopsies diagnosing DCIS during 2008–2014, were selected for re-examination of the original mammography and pathology data. For this sub-study, the mammography image(s) preceding the preoperative biopsy were reviewed by two experienced blinded radiologists and the pathology specimen(s) from the preoperative biopsy were reviewed by two experienced blinded breast cancer pathologists, each entering data into pre-defined data collection forms. To examine the potential influence of inter-observer variability, the preoperative biopsy DCIS grades from the pathology records were tabulated against the grades following review. In all cases, the original radiological and pathological data was used in the main analysis.

#### Subgroup analysis in patients eligible for trials

A subgroup analysis of upstaging and upgrading was performed in women who would have been eligible for the COMET [8], LORIS [9] and LORD [10] trials, based on their published eligibility criteria and using the information available in our cohort: age, screening mammography findings, biopsy method, DCIS grade on preoperative biopsy, symptomatic status at time of clinical examination and previous breast cancer history.

### Statistical methods

Confidence intervals for percentages were based on the normal approximation to the binomial distribution. Fisher exact tests of heterogeneity (for factors with 3+ categories) or difference (for factors with two categories) were conducted to identify associations between risk of upstaging or upgrading and different patient, tumour and radiological characteristics. As only preoperative biopsies showing low or intermediate grade could be upgraded to a higher DCIS grade, biopsies showing high or unknown grade were excluded from the analysis of factors associated with upgrading, but were included in the analysis of factors associated with upstaging. Risk factors for upstaging and upgrading were compared using logistic regression and significance tests for heterogeneity/difference used the likelihood ratio. Calculations were performed using Stata statistical software version 13.0 (StataCorp, College Station, TX). For completeness, unknown values are shown in the tables, but they were excluded from all the analyses, including the calculation of percentages.

## Results

**Fig. 1:**
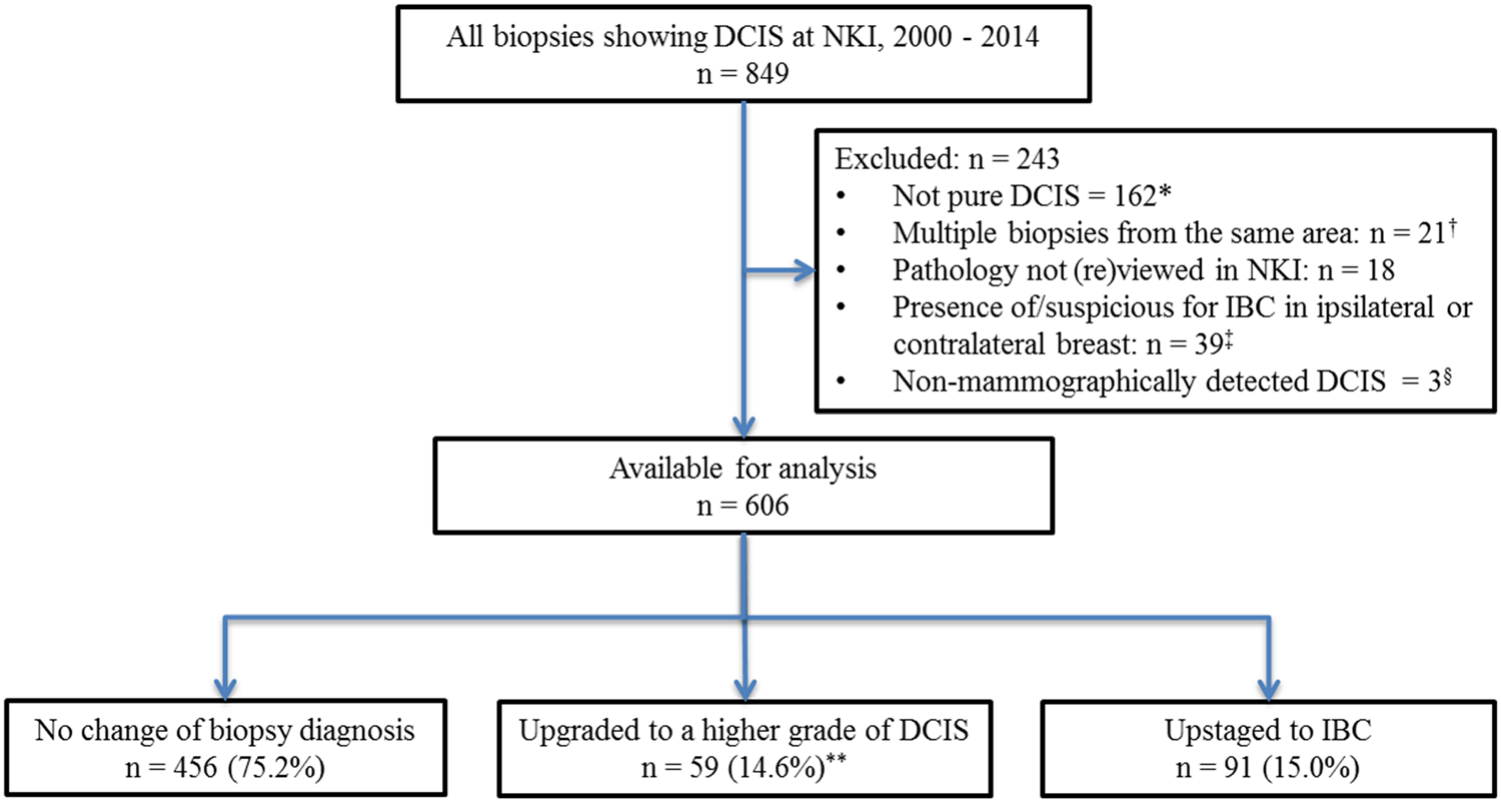
Derivation of the population used in the analysis. DCIS: Ductal carcinoma in situ, NKI: Netherlands Cancer Institute—Antoni van Leeuwenhoek hospital, IBC: Invasive Breast Cancer. *This category comprised 101 cases involving concomitant invasive breast cancer in the same biopsy, 22 cases involving lobular carcinoma in situ, 2 cases including Paget’s diseases, 24 cases with uncertain pathology and 13 cases with benign pathology. ^†^Where multiple biopsies were obtained from the same area within the breast, the highest grade was included. ^‡^In this category, 33 biopsies were undertaken in cases clinically suspicious for or proven to have invasive breast cancer, 4 biopsies were undertaken in cases of synchronous IBC in the contralateral breast for which women received neoadjuvant chemotherapy, and 2 biopsies were undertaken in cases where lymph node metastases were found although no breast invasion on final pathology was seen. ^§^In 3 cases, DCIS was detected only following magnetic resonance imaging. **Preoperative biopsies showing grade 3 (*n* = 191) or unknown grade (*n* = 10) could not be upgraded to a higher grade of DCIS, so the percentage upgraded was 14.6% (59/405)

In total, 849 biopsies showing DCIS were identified at NKI–AVL during 2000–2014. Our predetermined criteria excluded 243, leaving 606 biopsies in the study (Fig. 1). The number of women diagnosed with DCIS at NKI–AVL increased from 75 during 2000–2004, to 200 during 2005–2009, and to 331 during 2010–2014 (Table 1). 32.3% of women with DCIS were aged 20–49 years, 40.8% were 50–59 years, 19.6% were 60–69 years, and 7.3% were aged 70 + years. 53.6% of DCIS cases were screen-detected. Among those for whom information was available, 65.1% were diagnosed using 9G vacuum-assisted biopsy and 34.9% using 14G core-needle biopsy. 25.6% had low grade on biopsy, 41.3% had intermediate grade and 31.5% had high grade. All women underwent surgical excision, with approximately half receiving breast conserving surgery (52.8%).

**Table 1:** Characteristics of women diagnosed with ductal carcinoma in situ on preoperative biopsy and of their biopsies

Characteristics	Number of women/biopsies	(%)
Calendar year of diagnosis		
2000–2004	75	(12.4)
2005–2009	200	(33.0)
2010–2014	331	(54.6)
Age at diagnosis (years)		
20–49	196	(32.3)
50–59	247	(40.8)
60–69	119	(19.6)
70 +	44	(7.3)
Method of presentation		
Screen-detected^a^	324	(53.6)
Other^b^	280	(46.4)
Unknown	2	–
Method of biopsy		
Vacuum assisted biopsy (9G)	237	(65.1)
Core-needle biopsy (14G)	127	(34.9)
Unknown	242	–
Grade on preoperative biopsy		
Low	155	(26.0)
Intermediate	250	(42.0)
High	191	(32.0)
Unknown	10	–
Type of curative surgery		
BCS	224	(37.0)
BCS + SLNB	96	(15.8)
Mastectomy	76	(12.5)
Mastectomy + SLNB	206	(34.0)
Mastectomy + SLNB + AC	4	(0.7)
Total number of women/biopsies	606	(100)

*BCS* breast conserving surgery, *SLNB* sentinel lymph node biopsy, *AC* axillary clearance

^a^Including 275 diagnosed through the breast screening programme and 49 diagnosed during follow-up for family history/genetic predisposition

^b^Including 143 diagnosed during follow-up for a previously treated breast lesion, 74 presenting symptomatically, and 63 referred for routine second opinion

### Risks of upstaging and upgrading

Overall, 91 biopsies, i.e. 15.0% (95% confidence interval 12.3–18.1), were upstaged from DCIS on the biopsy to IBC in the final excised specimen (Table 2). A further 59, i.e. 14.6% (95% confidence interval 11.3–18.4), had a higher grade of DCIS in the final excised specimen compared with their initial biopsy (i.e. were upgraded). Of the 59 upgraded biopsies, 37 were intermediate grade on biopsy and high grade on final excised specimen, 20 were low grade on biopsy and intermediate grade on final excised specimen, and 2 were low grade on biopsy and high grade on final excised specimen.

**Table 2:** Numbers of preoperative biopsies of DCIS and final diagnosis following evaluation of surgically excised specimen by various characteristics

Characteristic	Final diagnosis from surgically excised specimen						
	Total number of biopsies	Number upstaged to IBC (%)	*p* for upstage versus no change^†^	Total number of biopsies eligible for upgrading*	Number upgraded to a higher grade of DCIS (%)	*p* for upgrade versus no change^†^	*p* for upgrade versus upstage^†^
Age at diagnosis (years)							
20–49	196	35(17.9)	0.43	131	21 (16.0)	0.09	0.21
50–59	247	35 (14.2)		169	29 (17.2)		
60–69	119	17 (14.3)		78	5 (6.4)		
70 +	44	4 (9.1)		27	4 (14.8)		
Method of biopsy							
9G VAC	237	26 (11.0)	0.07	171	12 (7.0)	< 0.001	0.14
14G Core-needle	127	21 (16.5)		79	18 (22.8)		
Unknown	242	44		155	29		
Mammographic lesion size (mm)							
0–19	180	15 (8.3)	0.004	145	15 (10.3)	0.01	0.53
20–50	144	27 (18.8)		77	12 (15.6)		
≥ 50	99	19 (19.2)		63	16 (25.4)		
Unknown	183	30		120	16		
Pathological lesion size (mm)							
0–19	201	14 (7.0)	<0.001	151	14 (9.3)	< 0.001	0.07
20–50	181	37 (20.4)		106	26 (24.5)		
≥ 50	107	21 (19.6)		62	16 (25.8)		
Unknown	117	19		86	3		
Mammographic lesion type							
Microcalcifications only	490	68 (13.9)	0.08	327	45 (13.8)	0.33	0.87
Other	97	20 (20.6)		65	12 (18.5)		
Unknown	19	3		13	2		
Radiological BIRADS score							
≤ 4	323	4 (12.7)	0.02	242	25(10.3)	0.03	0.54
5	47	12 (25.5)		19	5 (26.3)		
Other	236	38		144	29		
Method of presentation							
Screen-detected	324	41 (12.7)	0.35	229	34 (14.8)	0.62	0.32
Symptomatic	74	13 (17.6)		41	4 (9.8)		
Other^a^	208	37		135	21		
Presence of symptoms at examination^b^							
Symptoms	175	35 (20.0)	0.02	99	14 (14.1)	0.86	0.12
No symptoms	223	25 (11.2)		156	25 (16.0)		
Unknown	208	31		150	20		
Presence of palpable lump							
Yes	114	24 (21.1)	0.04	64	11 (17.2)	0.31	0.81
No	454	59 (13.0)		315	42 (13.3)		
Unknown	38	8		26	6		
Presence of necrosis on preoperative biopsy							
Yes	208	38 (18.3)	0.09	97	32 (33.0)	< 0.001	< 0.001
No	370	52 (14.1)		285	27 (9.5)		
Unknown	28	1		23	0		
Preoperative biopsy grade^c^							
1-Low	155	16 (10.3)	0.17	155	22 (14.2)	0.66	0.46
2-Intermediate	250	42 (16.8)		250	37 (14.8)		
3-High	191	30 (15.7)		–	–		
Unknown	10	3		0	0		
Total 606 (%, 95% confidence interval)	606	91 (15.0,12.3-18.1)		405*		59 (14.6, 11.3–18.4)	

*DCIS* ductal carcinoma in situ, *IBC* invasive breast cancer, *BIRADS* breast imaging reporting and data system, *VAC* vacuum assisted biopsy

*As only preoperative biopsies showing grade 1 or 2 could be upgraded to a higher grade of DCIS, preoperative biopsies showing grade 3 (*n* = 191) or unknown grade (*n* = 10) were not included in the analysis of factors associated with upgrading, but were included in the analysis of factors associated with upstaging

^†^Unknown values were omitted from tests of association

^a^Includes 143 biopsies undertaken during breast surgical follow-up, 63 undertaken for second opinion and 2 unknowns

^b^Any symptoms in either ipsilateral or contralateral breast

^c^Of the 22 low grade DCIS preoperative biopsies that were upgraded based on the surgically excised specimen 20 were upgraded to intermediate grade DCIS and 2 were upgraded to high grade DCIS

There was no significant association between age at diagnosis and risk of upstaging (*p*_heterogeneity_ = 0.43) or upgrading (*p*_heterogeneity_ = 0.09). The effect of biopsy method on upstaging was not significant (9G vacuum-assisted: 11.0% upstaged, 14G core-needle: 16.5% upstaged, *p*_difference_ = 0.07), but fewer 9G vacuum-assisted biopsies than 14G core-needle biopsies were upgraded (9G vacuum-assisted: 7.0% upgraded, 14G core-needle: 22.8% upgraded, *p*_difference_ < 0.001). Lesions measuring ≥ 20 mm on mammogram were more likely to be upstaged than smaller lesions (≥ 50 mm: 19.2% upstaged, 20–50 mm: 18.8% upstaged, 0–19 mm: 8.3% upstaged, *p*_heterogeneity_ = 0.004), while the risk of upgrading increased progressively with lesion size (≥ 50 mm: 25.4% upgraded, 20–50 mm: 15.6% upgraded, 0–19 mm: 10.3% upgraded, *p*_heterogeneity_ = 0.01). Lesions measuring ≥ 20 mm following final pathological analysis of the surgically excised specimen were more likely to be upstaged than smaller lesions (≥ 50 mm: 19.6% upstaged, 20–50 mm: 20.4% upstaged; 0–19 mm: 7.0% upstaged, *p*_heterogeneity_ < 0.001) and more likely to be upgraded (≥ 50 mm: 25.8% upgraded, 20–50 mm: 24.5% upgraded, 0–19 mm: 9.3% upgraded; *p*_heterogeneity_ < 0.001).

There was no significant association between presence on mammography of microcalcifications only compared with other abnormalities for risk of a biopsy being upstaged (13.9% vs 20.6%, *p*_difference_ = 0.08) or upgraded (13.8% vs 18.5%, *p*_difference_ = 0.33). Lesions with mammographic ‘Breast Imaging Reporting and Data System’ (BIRADS) score [19] of 5 were more likely to be upstaged than those with lower BIRADS scores (BIRADS score 5: 25.5% upstaged, BIRADS score ≤ 4: 12.7% upstaged, *p*_difference_ = 0.02) and also more likely to be upgraded (BIRADS score 5: 26.3% upgraded, BIRADS score ≤ 4: 10.3% upgraded, *p*_difference_ = 0.03). There were no significant differences between diagnosis via mammographic breast screening and symptomatic presentation for risk of upstaging (12.7% vs 17.6%, *p*_difference_ = 0.35) or upgrading (14.8% vs 9.8%, *p*_difference_ = 0.62). Presence of symptoms during clinical examination prior to biopsy increased risk of upstaging (20.0% vs 11.2%, *p*_difference_ = 0.02), but was not associated with risk of upgrading (14.1% vs 16.0%), *p*_difference_ = 0.86). The majority of women with symptoms had a palpable lump on examination, and presence of a palpable lump also increased risk of upstaging (21.1% vs 13.0%, *p*_difference_ = 0.04), but was not associated with the risk of a biopsy being upgraded (17.2% vs 13.3%, *p*_difference_ = 0.31).

Presence of necrosis on biopsy was not significantly associated with upstaging (18.3% vs 14.1%, *p*_difference_ = 0.09), but increased risk of upgrading (33.0% vs 9.5%, *p*_difference_ < 0.001) and presence of necrosis increased risk of upgrading more than risk of upstaging (*p*_difference_ < 0.001). Higher grade of DCIS on biopsy was not associated with being upstaged (*p*_difference_ = 0.17) or upgraded (*p*_difference_ = 0.66). There was no significant association between risk of either upstaging or upgrading of a biopsy and any of the other factors studied (Supplemental Table 2). If biopsies with the factors associated with disease underestimation, i.e. core-needle biopsy use, mammographic size > 20 mm, BIRADS score 5, presence of symptoms at examination, palpable lump, and presence of necrosis, are excluded, only 15 of the remaining 155 biopsies were upstaged (9.7%, 95% confidence interval 6.9–14.3) and only 5 of the remaining 127 biopsies were upgraded (3.9% (95% confidence interval 0.6–7.3).

### Interobserver variability in biopsy grade

No preoperative biopsies were upstaged to IBC following blinded re-review of 159 biopsies, but there was interobserver variability for DCIS grade on preoperative biopsy (Table 3). Amongst 36 biopsies initially rated as low grade DCIS, none were upgraded to high grade DCIS but 9 (25%) were upgraded to intermediate grade DCIS. Amongst 71 biopsies initially rated as intermediate grade DCIS, 10 (14.1%) were upgraded to high grade DCIS.

**Table 3:** Outcome of independent review of preoperative biopsies: numbers of biopsies by initial DCIS grade and DCIS grade following re-review by an independent blinded pathologist

Initial biopsy grade	Biopsy grade re-review by independent blinded pathologist (%)					
	1-Low	2-Intermediate	3-High	Total reviewed	Not reviewed	Overall total
1-Low	27 (75.0)^a^	9 (25.0)	0	36 (100.0)	119	155
2-Intermediate	10 (13.7)	51 (69.9)^a^	10 (14.1)	71 (100.0)	179	250
3-High	1 (2.0)	19 (37.3)	31 (60.8)^a^	51 (100.0)	140	191
Unknown	0	1	0	1	9	10
Total	38 (23.9)	80 (50.3)	41 (25.8)	159 (100.0)	447	606

*DCIS* ductal carcinoma in situ

^a^Cells where the initial biopsy grade was identical to that reported following independent blinded re-review by an experienced pathologist

### Characteristics of upstaged cases

Of the 91 upstaged biopsies, information on IBC grade was available in 82 (Table 4). Of these, the IBC was low grade in 41.5% (34/82) cases, intermediate grade in 37.8% (31/82) cases, and high grade in 20.7% (17/82) cases. All 16 biopsies showing low grade DCIS that were subsequently upstaged to IBC, also had low grade IBC. This pattern was not observed in biopsies that showed intermediate or high grade DCIS which, if upstaged, resulted in any of the three grades of IBC. Information on the size of the IBC was available for 88 of the 91 upstaged cases. The invasive component was < 10 mm in 87.5% (77/88) of the upstaged biopsies, and was < 20 mm in all 88. There was no clear association between IBC grade and IBC size. Information on oestrogen receptor (ER) expression within the IBC was available for 82 of the 91 upstaged cases, and 85.4% (70/82) were ER positive while 14.6% (12/82) were ER negative.

**Table 4:** Characteristics of the 91 upstaged biopsies

	Grade of IBC component in upstaged cases				Total
	1-Low	2-Intermediate	3-High	Unknown	
Grade of DCIS on preoperative biopsy					
1-Low	16	0	0	0	16
2-Intermediate	13	19	7	3	42
3-High	4	12	8	6	30
Unknown	1	0	2	0	3
Total pathological size of IBC component (mm)					
< 5	14	16	4	6	40
5–9	15	11	9	2	37
10–14	2	3	1	0	6
15–20	1	1	3	0	5
Unknown	2	0	0	1	3
Oestrogen receptor expression in the IBC component					
Negative	0	3	7	2	12
Positive	33	28	10	7	78
Unknown	3	3	1	2	9
Total	34	31	17	9	91

Association between the grade of the invasive breast cancer component and the grade of DCIS on preoperative biopsy (upper panel), and between the size and the grade of the invasive component amongst upstaged biopsies (lower panels)

*DCIS* ductal carcinoma in situ, IBC: invasive breast cancer

### Subgroup analysis in patients eligible for the randomised trials

When the strict eligibility criteria used in the three ongoing randomised trials of non-operative treatment of biopsies showing DCIS were applied, only a small number from our cohort would have been eligible for randomisation in the LORD trial (12/606), but greater numbers were eligible in the other two (LORIS: 68/606, COMET: 57/606) (Supplemental Table 3). When compared with the 15.0% (91/606) of biopsies upstaged in our cohort, the application of the trial eligibility criteria reduced the percentage of biopsies being upstaged in both trials with reasonable numbers of eligible women: LORIS: 10.3% (7/68), COMET: 10.5% (6/57). However, compared with the 14.6% (59/405) upgraded in our cohort, the application of trial eligibility criteria had the opposite effect in both trials: LORIS: 19.1% (13/68), COMET: 15.8% (9/57). For the LORD trial, of the 12 eligible women, 2 biopsies were upstaged and 1 upgraded.

## Discussion

This study demonstrates that the risk of a diagnosis of DCIS on preoperative biopsy being upstaged to one of IBC after examination of the excised specimen is around 15%. This is considerably lower than that suggested by previous studies, and the difference is likely to be due to careful identification and exclusion of women already known to be at increased risk of IBC. In this respect our study is more comparable with women currently being considered for non-operative management than previous reports. Our study, which is one of the largest to date, has also examined risk factors for upstaging and upgrading of preoperative biopsies in women diagnosed with DCIS. The risk of upstaging and/or upgrading was found to be increased with use of vacuum-assisted biopsy, large mammographic and pathological lesion size, high mammographic BIRADS score, presence of symptoms such as a palpable lump on examination, and presence of necrosis on biopsy. Consideration of these factors should aid risk stratification of women with DCIS being considered for non-operative management.

Several of the above factors are already included in the eligibility criteria used to select women for inclusion in the ongoing trials of non-operative management of DCIS [8–10]. We have, however, identified some additional factors that may be useful. The association between larger DCIS size on mammography and upstaging to IBC or upgrading to higher grade DCIS may be due to the heterogeneous growth patterns seen in larger DCIS lesions [20]. While IBC spreads through the ductal basement membrane, DCIS lesions usually grow along the milk ducts with a branched growth process, with emerging and competing cell-lines of low, intermediate and high-grade disease often resulting in a diverse cell-line environment [21, 22]. Thus, the larger the disease area, the higher the likelihood of IBC or more than one grade of DCIS being present that was not sampled during preoperative biopsy and only detected following pathological analysis of the surgically excised specimen [23].

Upgraded lesions were also more likely to have necrosis detected on preoperative biopsy compared with lesions with unchanged diagnosis following surgery. Necrosis in the DCIS biopsy tissue indicates that cells in the sampled area have died. The pathological finding of necrosis often accompanies rapid cell turnover, where the rate of growth may have outstripped the blood supply to a central portion of the cancer. The presence of this finding on biopsy, similar to large lesion size described above, raises the risk of a higher grade of DCIS being present.

There was strong evidence that fewer vacuum-assisted (9G) than core-needle (14G) biopsies were upgraded (*p*_difference_ < 0.001), while the effect of biopsy method on upstaging was not quite significant (*p*_difference_ = 0.07). Preoperative biopsy for breast cancer has conventionally been radiologically guided core-needle biopsy and, in more recent years, vacuum-assisted biopsy. Vacuum-assisted biopsy devices generally obtain more tissue than core-needle biopsy devices, increasing the likelihood of obtaining a representative tissue specimen [24]. This is the likely explanation for the lower proportion of vacuum-assisted than core-needle biopsies upgraded in the present study.

Our findings provide reassurance for both patients and clinicians regarding participation in ongoing randomised trials of non-operative management, especially for women with low grade DCIS on preoperative biopsy [15]. Additionally, in upstaged cases when IBC was found only following final pathology, it was often a small, low grade, ER+ tumour, which generally has excellent prognosis, even when detected during follow-up screening. A recurring concern impacting participation in the active surveillance trials has been interobserver variation amongst pathologists reporting preoperative biopsies [13, 25]. In our study no biopsies were upstaged to IBC and, whilst there was some interobserver variability in DCIS grade none were upgraded from low to high grade [26]. The retrospective application of the eligibility criteria of these ongoing trials in our cohort was also reassuring as regards risk of upstaging, but less clear for upgrading.

Although our study was limited to a single centre, the characteristics of the tumours, i.e. ‘case mix’, diagnosed and treated at this centre are similar to those seen at the national level in the Netherlands and many other countries [27]. Consequently, it is likely that our findings have wide applicability. To optimize risk stratification for DCIS and also to minimise risk of subsequent upstaging or upgrading, developments in genomics and candidate gene analysis of tumour tissue are ongoing [28, 29]. These developments can be expected to move diagnostic criteria away from traditional phenotypic characteristics, e.g. nuclear grade and growth pattern, as the chief determining factors of DCIS prognosis, towards novel biomarkers for progression to IBC, such as markers of proliferative signalling, hallmarks of genome instability, and micro-environmental factors [30]. Such approaches may, in the future, further improve the diagnostic accuracy of preoperative biopsy to detect occult high-risk lesions.

## Conclusions

Over the years there has been a trend towards less invasive surgical treatment of both IBC and DCIS. The publication of findings from three on-going randomised trials of non-operative treatment in DCIS are anticipated in the coming decade and they will determine whether a further paradigm shift in its treatment is appropriate. Our findings provide reassurance to healthcare providers and patients alike regarding participation in both ongoing and future trials of non-operative management of DCIS by demonstrating lower proportions of upstaged preoperative biopsies than previously reported, an absence of missed IBC from interobserver variation amongst pathologists reviewing preoperative biopsies, and favourable prognostic features amongst the minority of cases that were upstaged. While our findings confirm that method of biopsy and absence of symptoms at presentation are important eligibility criteria for such trials, they also suggest that age at diagnosis and mammographic morphology may be less important than currently thought. This may, in the future, provide scope for widening the eligibility of women for non-operative treatment without compromising risk of upstaging or upgrading. Our findings also show that presence of necrosis on biopsy and mammographic lesion size are more important than previously considered which may help to lower upstage and upgrade risks. Future interpretation and implementation of the findings from the on-going trials will need to take careful account of the factors determining biopsy accuracy, as described in the present study, to guide the path towards non-operative management for low-risk women with DCIS.

## Electronic supplementary material

Below is the link to the electronic supplementary material.

Supplementary material 1 (PDF 304 kb)

## Data Availability

The datasets generated during and/or analysed during the current study are not publically available due to participant confidentiality, but are available from the Netherlands Cancer Institute—Antoni van Leeuwenhoek Hospital on reasonable request and appropriate ethical approval.

## Abbreviations

BIRADS: Breast imaging reporting and data system
DCIS: Ductal carcinoma in situ
ER: Oestrogen receptor
IBC: Invasive breast cancer
NKI–AVL: Netherlands Cancer Institute—Antoni van Leeuwenhoek Hospital
