# Supplementary material for: Reliability of preoperative breast biopsies showing ductal carcinoma in situ and implications for non-operative treatment: a cohort study
Source: Breast Cancer Res Treat. 2019 Aug 6;178(2):409–18. doi: 10.1007/s10549-019-05362-1 (PMC6797705; doi:10.1007/s10549-019-05362-1)
Supplement: Supplementary file 1 — Supplementary material 1 (PDF 304 kb) [file 10549_2019_5362_MOESM1_ESM.pdf]

Online Supplementary Material

Supplementary Appendix 1: Search criteria used to identify biopsies for inclusion in study.

The process involved 3 searches:

Search 1:
PALGA database search terms: “Mamma * Aard (A) * Diagnose (D)”
Period: 2008 - 2014

With (A) containing the search terms
- ‘Biopsie’
- ‘Biopt’
- ‘Naaldbiopt’

And (D) containing the search terms
- ‘Ductaal carcinoma in situ’
- ‘Ductaal carcinoom in situ’
- ‘Carcinoma in situ’
- ‘Intraductaal carcinoom’
- ‘Intraduct carcinoma’
- ‘Intraduct carcinoom’
- ‘Intraductaal carcinoom in situ’
- ‘Intraductaal carcinoom niet infiltrerend’
- ‘Ductaal carcinoom insitu’
- ‘Ductus carcinoma in situ’
- ‘Duct carcinoma in situ’
- ‘Atypische ductale hyperplasie’
- ‘Ductaalcarcinoma in situ’

The final search term was therefore: Mamma AND [A1 OR A2 OR A3] AND [D1 OR D2 OR D3 etc]

Search 2:
- 2008 -2014: Only T-numbers (histology)
- 1e: ^mamma
- 2e: biop
- 3e: duct
- does not match: infiltrerend
- does not match: invasie

Search 3:
- 2008 - 2014: only T-numbers (histology)
- 1e: ^mamma
- 2e: biop
- 3e: situ
- does not match: duct
- does not match: lobu

This tiered-approach in searching PALGA resulted in a list with 1327 records (not unique patients). These records were then requested from the NKI-AVL hospital tumour registry and duplicates deleted.

Supplementary Table 1: Definitions used for ‘upstaged’, ‘upgraded’ and ‘no change’ and 'downgraded'.

Outcome	Initial biopsy →	Final histology
Upgraded	DCIS Grade 1	DCIS Grade 2
	DCIS Grade 1	DCIS Grade 3
	DCIS Grade 2	DCIS Grade 3
Upstaged	DCIS (any grade)	IBC (any grade)
	DCIS (any grade)	IBC (any grade) & DCIS (any grade)
	DCIS (any grade)	IBC (any grade) & DCIS (any grade) & LCIS (any grade)
No Change	DCIS Grade 1	DCIS Grade 1*
	DCIS Grade 2	DCIS Grade 2
	DCIS Grade 3	DCIS Grade 3
	DCIS (any grade)	LCIS (any grade)
	DCIS (any grade)	DCIS (any grade) & LCIS (any grade)
Downgraded[†]	DCIS Grade 3	DCIS Grade 2
	DCIS Grade 3	DCIS Grade1
	DCIS Grade 2	DCIS Grade 1*

*In cases where DCIS grade 1 was clearly seen on preoperative biopsy but only atypical ductal hyperplasia (ADH) was seen in the excised specimen, the excised specimen was referred to as DCIS grade 1. [†] ‘Downgraded’ cases were grouped with ‘No change’ during the analysis of risk factors for upgrading and upstaging of preoperative biopsies.

Supplementary Table 2: Numbers of preoperative biopsies of DCIS by final diagnosis following evaluation of surgically excised specimen for various additional characteristics.

Characteristic	Final diagnosis from surgically excised specimen					
	Total number of biopsies	Upstaged to IBC (%)	P for upstage vs no change†	Total number of biopsies*	Upgraded to a higher grade of DCIS (%)	P for upstage vs no change†
**Calendar year of diagnosis**						
2000 - 2004	75	12 (16·0)	0·65	39	9 (23·1)	0·09
2005 - 2009	200	26 (13·0)		120	22 (18·3)	
2010 - 2014	331	53 (16·0)		246	28 (11·4)	
**Mammographic location (quadrant)**						
Upper inner	43	8 (18·6)	0·43	28	3 (10·7)	0·21
Upper outer	281	43 (15·3)		193	27 (14·0)	
Lower inner	59	6 (10·2)		40	8 (20·0)	
Lower outer	26	1 (3·8)		17	2 (11·8)	
Upper central	33	8 (24·2)		23	5 (21·7)	
Lower central	15	3 (20·0)		10	0 (0·0)	
Inner central	16	2 (12·5)		9	2 (22·2)	
Outer central	27	5 (18·5)		19	0 (0·0)	
Retroareolar	58	6 (10·3)		32	8 (25·0)	
Unknown	48	9		34	4	
**Mammographic calcification distribution**						
Diffuse	32	3 (9·4)	0·09	25	5 (20·0)	0·35
Regional	1	1 (100·0)		0	0 (-)	
Grouped	266	29 (10·9)		191	23 (12·0)	
Linear	28	5 (17·9)		17	3 (17·6)	
Segmental	56	10 (17·9)		31	6 (19·4)	
Unknown	223	43		141	22	
**Mammographic mass shape**						
Round	4	0 (0·0)	0·50	3	1 (33·3)	0·66
Oval	12	4 (33·3)		10	0 (0·0)	
Irregular	9	1 (11·1)		6	1 (16·7)	
Unknown	581	86		386	57	
**Menopausal status**						
Premenopausal	216	35 (16·2)	0·53	150	23 (15·3)	0·64
Postmenopausal	303	44 (14·5)		200	27 (13·5)	
Unknown	87	12		55	9	
**Lesion laterality**						
Left	306	52 (17·0)	0·30	199	23 (11·6)	0·12
Right	300	39 (13·0)		206	36 (17·5)	
**Previous breast lesions**						
Yes‡	94	13 (13·8)	0·87	62	9 (14·5)	1·00
No	512	78 (15·2)		343	50 (14·6)	
**Immunohistochemistry performed on biopsy**						
Yes	106	14 (13·2)	1·00	84	12 (14·3)	0·29
No	288	41 (14·2)		187	18 (9·6)	
Unknown	212	36		134	29	
**Fine-needle aspiration**						
Yes	149	29 (19·5)	0·32	105	16 (15·2)	0·36
No	111	17 (15·3)		73	7 (9·6)	
Unknown	346	45		227	36	
**Total (%, 95% confidence interval)**	606	91 (15·0, 12·3-18.1)		405*	59 (14·6, 11.3-18.4)	

BIRADS: Breast Imaging Reporting and Data System. *As only preoperative biopsies showing grade 1 or 2 could be upgraded to a higher grade of DCIS, preoperative biopsies showing grade 3 (n=191) or unknown grade (n=10) were not included in the analysis of factors associated with upgrading, but were included in the analysis of factors associated with upstaging. † Unknown values were omitted from tests of association. ‡Includes 58 contralateral invasive breast cancer, 31 contralateral ductal carcinoma in situ (DCIS), 2 contralateral lobular carcinoma in situ (LCIS), 1 ipsilateral invasive breast cancer, 1 ipsilateral DCIS, 1 ipsilateral LCIS.

Supplementary Table 3: Retrospective application of the eligibility criteria of the three on-going randomised trials of non-operative management of low-risk DCIS to the present cohort and the effect of these criteria on the number of biopsies upstaged and upgraded.

Criteria applied	Total meeting criteria	Unchanged	Upstaged to IBC	Upgraded to a higher grade of DCIS
Current cohort	606	456	91	59
LORIS trial*	68	48	7	13
COMET trial†	57	42	6	9
LORD trial‡	12	9	2	1

DCIS: ductal carcinoma in situ*;* LORIS: Surgery versus Active Monitoring for Low Risk Ductal Carcinoma In Situ (DCIS)[1]; COMET: Comparison of Operative to Monitoring and Endocrine Therapy Trial For Low Risk DCIS[2]; LORD: LOw Risk DCIS study[3].

*The LORIS study criteria applied to the current cohort limited the inclusion criteria to women aged ≥46; those with screening mammography findings showing calcifications only; biopsy undertaken by vacuum assisted biopsy or core biopsy; preoperative biopsies showing low or intermediate grade DCIS; no symptoms at time of presentation. All patients with a previous breast cancer history, i.e. previous diagnosis of either ipsilateral/contralateral invasive breast cancer, DCIS or LCIS, were excluded. Information on familial breast cancer risk or on prior exposure to mantle field radiotherapy was not available so could not be applied.

†The COMET study criteria applied to the current cohort limited the inclusion criteria to women aged ≥40; those with screening mammography findings showing calcifications only; biopsy undertaken by vacuum assisted biopsy or core biopsy; preoperative biopsies showing low or intermediate grade DCIS; no symptoms at time of presentation. All patients with a previous breast cancer history, i.e. previous diagnosis of either ipsilateral/contralateral invasive breast cancer, DCIS or LCIS, were excluded. Information on prior chemoprevention and DCIS ER, PR, and HER2 status was not available so could not be applied.

‡The LORD study criteria applied to the current cohort limited the inclusion criteria to women aged ≥45; those with screening mammography findings showing calcifications only; biopsy undertaken by vacuum assisted biopsy only; solely preoperative biopsies showing low grade DCIS; no symptoms at time of presentation. All patients with a previous breast cancer history, i.e. previous diagnosis of either ipsilateral/contralateral invasive breast cancer, DCIS or LCIS, and those with bilateral lesions at presentation were excluded.

References for Supplementary Table 3

1. Francis A, Thomas J, Fallowfield L, et al. Addressing overtreatment of screen detected DCIS; the LORIS trial. European Journal of Cancer 2015; 51(16): 2296-303.
2. Youngwirth LM, Boughey JC, Hwang ES. Surgery versus monitoring and endocrine therapy for low-risk DCIS: The COMET Trial. Bulletin of the American College of Surgeons 2017.
3. Elshof LE, Tryfonidis K, Slaets L, et al. Feasibility of a prospective, randomised, open-label, international multicentre, phase III, non-inferiority trial to assess the safety of active surveillance for low risk ductal carcinoma in situ – The LORD study. European Journal of Cancer 2015; 51(12): 1497-510.
